# Recruiting Black Men Who Have Sex With Men (MSM) Couples via Dating Apps: Pilot Study on Challenges and Successes

**DOI:** 10.2196/31901

**Published:** 2022-04-08

**Authors:** Yong Darin Witkovic, Hyunjin Cindy Kim, Darius Jovon Bright, Judy Y Tan

**Affiliations:** 1 Department of Psychology Palo Alto University Palo Alto, CA United States; 2 Division of Prevention Science Center for AIDS Prevention Studies University of California San Francisco San Francisco, CA United States

**Keywords:** African American, sexual and gender minorities, homosexuality, male, HIV, mHealth intervention, mobile applications, apps, sexual partners, investigative techniques, community engagement, MSM, Black men, mobile app, LGBT, research methods, recruitment, online dating, social network

## Abstract

**Background:**

HIV disproportionately impacts Black men who have sex with men (MSM), and targeting the primary relationship (ie, couples) using mobile technology for health holds promise for HIV prevention. Web-based recruitment of MSM is commonly employed in HIV prevention and intervention research. However, little known about recruiting Black MSM couples on the internet in the United States.

**Objective:**

This study describes the process of recruiting Black MSM couples over social networking and dating apps frequented by MSM. We describe the activities for recruiting, screening, and enrolling participants as part of a randomized trial employing a multipronged recruitment approach.

**Methods:**

Black MSM in couples were recruited via three apps (ie, *Jack’d*, *Adam4Adam*, and *Growlr*) between May 2020 and March 2021 during the COVID-19 pandemic in the United States. Black MSM couples were eligible if one or both partners are Black, MSM, and living with HIV, and if both partners were 18 years or older, and have been together for at least 2 months in what they both consider a primary relationship (ie, one in which both partners reported feeling most committed to over any other partner or relationship).

**Results:**

A total of 10 Black MSM couples (n=20) were enrolled via social networking apps. App recruitment activities were a combination of passive (eg, in-app advertisements) and active (eg, direct messaging of users) engagement. Recruitment approaches varied by the social networking app owing to differences in app features. A full-time recruiter experienced challenges such as bugs (ie, technical errors in computer program or system), navigating technical requirements specific to each app, and web-based harassment.

**Conclusions:**

Despite challenges, it was possible to recruit Black MSM couples virtually into research as part of a multipronged recruitment strategy. We identify tips for using web-based dating and other social networking apps as part of a recruitment strategy in future research with Black MSM couples.

## Introduction

HIV remains a global health issue requiring continued efforts in prevention and intervention in low-, middle-, and high-income countries [1,2]. Within the United States, HIV disproportionately affects men who have sex with men (MSM). This health disparity is even greater among Black or African American (hereafter “Black”) men [3-6]. Half of all Black MSM are estimated to acquire HIV in their lifetimes compared to one in 11 for White MSM [7]. The primary romantic relationship is an intervention target given high rates of seroconversion among MSM in these relationships [8-11]. Among Black MSM, nationwide estimates indicate that one-third to a half of those with HIV are in a primary relationship [12-14]. Therefore, research on couples remains crucial in HIV/AIDS prevention and intervention.

Location-based dating and social networking apps have become an option for participant recruitment in HIV/AIDS and sexual health research [15-17]. The advantages of app-based recruitment in HIV research are recently highlighted by the COVID-19 pandemic whereby in-person recruitment was prohibited owing to physical distancing and other public health measures [18]. Recruiting couples on the internet requires special consideration for relationship verification and data validation [19-21]. Emergent studies have used apps and other social media (eg, Facebook and Instagram) to recruit MSM couples [22,23]. However, knowledge gaps exist for using dating and social networking apps to engage racial or ethnic minority MSM couples.

Recruiting Black MSM couples into research studies presents important considerations and is challenging for myriad reasons. Distrust of research and medical institutions and cultural stigma concerning race, sexual orientation, and HIV status are barriers to research participation for Black MSM [24,25]. MSM in couples may have a diverse range of agreements regarding sex with others outside of their relationship. Sexual agreements are the mutual understanding between primary partners regarding what sexual behaviors are allowed [26]. These agreements are prevalent among 58% to 99% of same-sex male couples [27], with 11% to 64% of these agreements including sex with outside partners [27]. Given that some MSM have agreements regarding outside partners, dating and social networking apps provide a way to reach partnered MSM that may use apps to socialize or find potential sexual partners.

Few studies have presented details on using dating and social networking apps to recruit Black MSM couples, highlighting a potential knowledge gap on methods for engaging couples into research. Thus, the goal of this study is to describe the process of using dating and social networking apps to recruit same-sex couples to inform future trial designs. This study is part of a multipronged recruitment approach of a pilot randomized controlled trial (RCT) with Black MSM couples with HIV.

## Methods

### Study Overview

The dating app recruitment process described herein was part of a multipronged recruitment approach of a pilot RCT to test the feasibility and acceptability of a mobile app intervention for improving HIV care and treatment among Black MSM couples living with HIV. We targeted recruitment efforts on dating and social networking apps frequented by Black MSM: *Jack’d*, *Adam4Adam* (*A4A*), and *Growlr* [19,28-31]. Qualitative data particular to each app are described below to highlight the unique success and challenges experienced using this underutilized recruitment approach. To maximize engagement with Black MSM, we hired a Black, cisgender, same-gender-loving–identified man as the study recruiter who performed all recruitment activities and documented the recruitment process. Recruitment occurred between May 2020 and March 2021. Black MSM couples were eligible if one or both partners are Black, MSM, and living with HIV, if both partners were 18 years or older, and have been together for at least 2 months in what they both consider a primary relationship (ie, one in which both partners reported feeling most committed to over any other partner or relationship).

Owing to differences in user engagement requirements by app, we used different engagement approaches by app. On *Jack'd*, recruitment was conducted through their in-app advertisements. Interested candidates who clicked on the advertisement were directed to a Qualtrics prescreener questionnaire that obtained basic qualifying information (eg, current place of resident, race, relationship and HIV status, and length of time on antiretroviral medications for HIV). Study staff then contacted eligible candidates via SMS text message using the telephone number provided.

Recruitment on *A4A* was carried out by sending private SMS text messages to potential participants using the in-app messaging feature. The recruiter identified potential participants using the app’s search filters which allowed users to filter through other users’ profiles on the basis of set criteria such as their race, HIV status, and relationship status. The study recruiter identified users whose race or ethnicity was set to Black, African American, or mixed. We included “mixed” race because many Black MSM may identify as mixed race given the diversity among Black communities. We also found that some users identify their race as mixed to avoid being filtered out by users who filter out Black-identified users within the app. The study recruiter identified users whose HIV status was set to HIV-positive, undetectable, or unanswered. Users who left their HIV status unanswered were considered for the study as it would encompass anyone who has never been tested or chose not to disclose their serostatus on the internet. Finally, the study recruiter identified users whose relationship status was set to dating, partnered, open relationship, polyamorous, or married.

Once potential participants were identified, the study recruiter privately messaged each individually. *A4A* has a message delivery report in its platform, which allowed recruiters to know if a message has been read or not. Users who read but did not respond within 48 hours of the first message being sent were sent a follow-up message asking if they were still considering participation in the study or were no longer interested. The messages that remained unread would require no follow-up as those users were likely inactive. Users who communicated interest then were asked to complete a phone screener with a study staff to determine eligibility.

Recruitment on *Growlr* was carried out by sending private messages to potential participants using the in-app messaging feature and in-app advertisements contained the weblink to a Qualtrics prescreener. A *Growlr* paid service, the “SHOUT!” feature, allowed the recruiter to send the study information to multiple people in a specified vicinity.

A total of 10 couples (N=20) recruited via apps were enrolled in the trial, including 7 same-race Black couples and 3 interracial couples.

### Ethical Considerations

This study received ethics approval from the institutional review board of University of California, San Francisco (IRB#15-18042). All participants provided informed consent to participate in the study.

## Results

### Results Overview

Individual- and couple-level characteristics of the couples recruited via apps are reported in Tables 1 and 2, respectively. The following outlines findings resulting from the process of recruiting participants via each app.

**Table 1:** Individual-level participant demographic characteristics of couples recruited from dating apps (N=20).

Characteristics			Values
Age (years), mean (SD), range			36 (13), 20-54
Length of relationship (months), mean (SD), range			5.7 (9.3), 2-336
**Ethnicity, n (%)**			
	Hispanic or Latino	3 (15)	
	Not Hispanic or Latino	17 (85)	
**Race, n (%)**			
	African American or Black	15 (75)	
	White	3 (15)	
	More than one race	2 (10)	
**Serostatus, n (%)**			
	Living with HIV	15 (75)	
	Not living with HIV	5 (25)	
**Cohabitation, n (%)**			
	Living together	16 (80)	
	Not living together	4 (20)	

**Table 2:** Couple-level characteristics by HIV serostatus and race (N=20).

Status	Same-race participants, n	Interracial participants, n	Couples, n (%)
Seroconcordant-positive (both members are HIV-positive)	4^a^	1	5 (50%)
Serodiscordant (one partner with an HIV-positive status and the other with an HIV-negative or unknown status)	3	2	5 (50%)
Total	7 (70%)	3 (30%)	10

^a^There was one serodiscordant-positive couple in which both partners are multiracial. They identify as African American or Black and another race (eg, Latinx and Native American).

### Jack’d

#### Overall Findings

In-app advertisements on *Jack’d* were used for recruitment on the platform. Eligible candidates who completed the Qualtrics prescreener questionnaire through the study advertisement and were contacted by recruiters via SMS text message with the telephone number they had provided. If the candidate did not respond to the SMS text message within 24 hours, a recruiter would follow up with a telephone call and leave a voicemail message if there was no answer. Potential candidates had 1 week to respond before another attempt to make contact was made. This pattern of correspondence continued until either the candidate indicated that he/she was no longer interested or the telephone number was no longer in service. Interested and eligible candidates who completed the Qualtrics prescreener would then complete a telephone screener. Participants were scheduled for an interview once they provide informed consent to participate.

A total of 35,912 unique impressions, or the number of times the study advertisement was displayed to a user for the first time, occurred on *Jack’d* in 4 major cities (Atlanta, Georgia; Los Angeles, California; Houston, Texas; and Washington, District of Columbia). Of these views, 924 users clicked on our advertisement at least once. Consequently, the click-through rate, or number of unique clicks divided by the number of unique impressions, ranged between 0.85% (Atlanta, Georgia) to 1.16% (Houston, Texas).

#### Character Limits for Advertisement Placement

**Figure 1:**
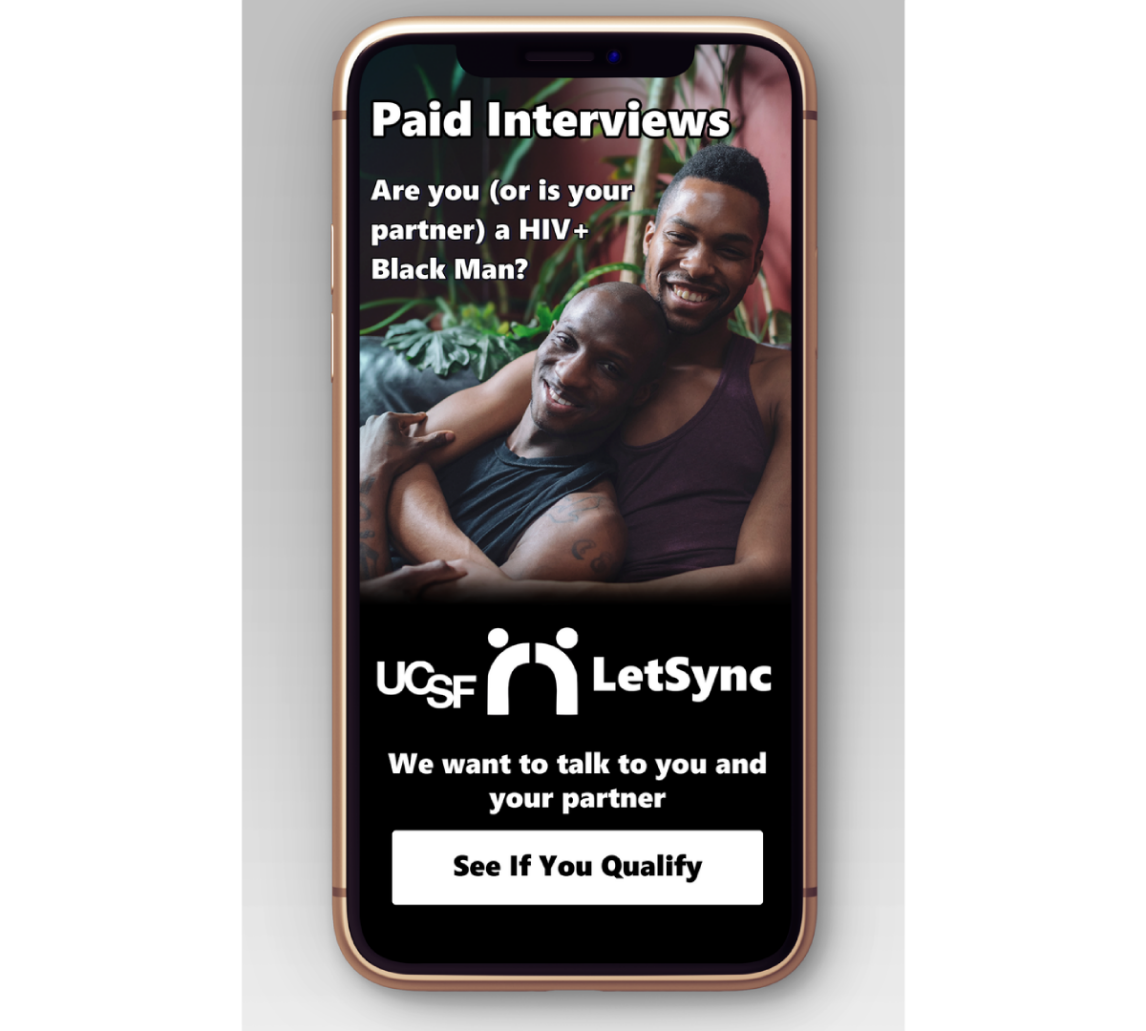
Inclusion of part of the study description into the image selected for our profile at an extra cost in the app interface.

**Figure 2:**
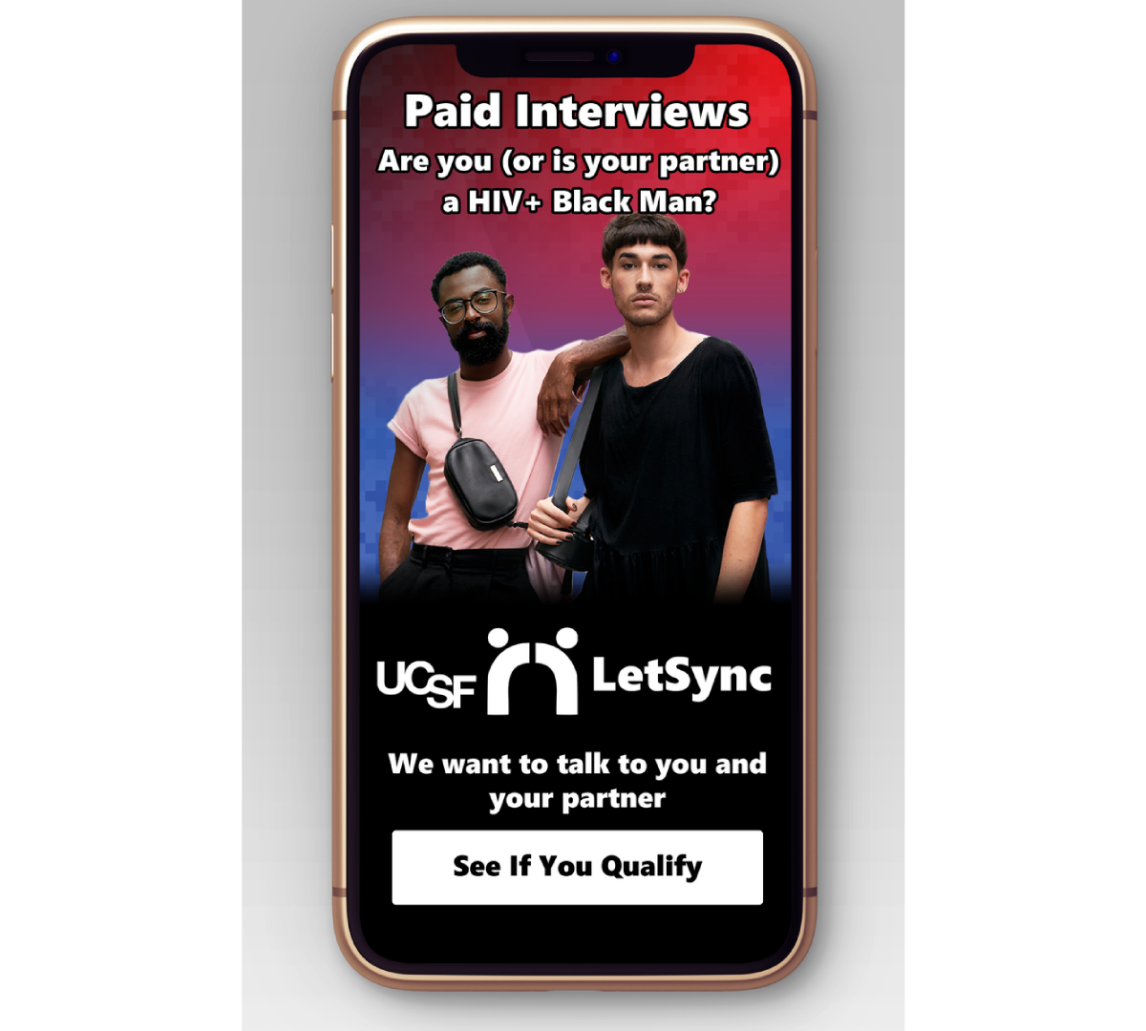
Screenshot of the image selected for our profile in the app.

Though recruitment on *Jack’d* was carried out through in-app advertisements, imposed character limits made it difficult to fully describe the target population and goals of the study. One solution was to include part of the study description into the image selected for our profile at an extra cost (Figures 1 and 2). *Jack’d* removed our advertisements and stated that adding more text to our recruitment advertisements would be an extra cost. Our team elected to pay the additional fee to include more description in our in-app advertisements so that interested applicants had more information prior to completing the prescreening measure.

### Adam4Adam

#### Technical Bugs

The recruiter experienced functionality issues with the web-based browser and mobile app versions of *A4A*. The web-based version was designed to look like the app, but there were technical bugs with several functions. For example, the recruiter made edits to the profile on the web-based version; however, these edits were not always reflected in the app version. Moreover, blocks of text from the recruiter profile would often be removed without notification or explanation, which would leave out key details of the study and regular monitoring would be required to ensure that information published to the app profile was not deleted by the app. Unfortunately, when information was deleted from the profile no error messages or warnings had been communicated to the recruiter. As such, there may have been times when potential candidates missed vital information about the study.

Potential candidates were contacted on the basis of their eligibility potential, which was determined by using app search filters (eg, candidate identified race, relationship, and HIV status). Additionally, recruiters scanned through details on their candidates’ profiles for information that may qualify or disqualify them for the study. A total of 292 potential candidates were contacted on *A4A* across 15 different states. Searches were conducted across all large geographical regions of the United States including the West (California, Nevada, Arizona, and Washington), Midwest (Ohio, Illinois, and Wisconsin), South (Mississippi, Texas, Georgia, Florida, North Carolina, and Tennessee), and Northeast (Massachusetts and New Jersey). Participants contacted in accordance with the state were as follows: Arizona (n=10), Georgia (n=17), Illinois (n=34), Massachusetts (n=7), Mississippi (n=14), Nevada (n=10), New Jersey (n=12), North Carolina (n=45), Ohio (n=19), Tennessee (n=18), Texas (n=25), Washington (n=9), and Wisconsin (n=3). Owing to an unexpected account suspension, we were unable to breakdown numbers between California and Florida (n=69).

#### Existence of Bots

Successful engagements with potential participants on *A4A* could be improved simply by the recruiter distinguishing themselves from automated “bot” profiles that function to send spam and are often ignored by app users. The recruiter found positive changes in user responses when he developed rapport with other users. For example, one user had a profile photo with a dog, prompting the recruiter to comment, “Cute dog, it reminds me of my childhood pet,” followed by a self-introduction. In another successful recruitment interaction, the recruiter started a conversation inquiring about the reference of a song in a user’s profile name. The shared knowledge between the user and recruiter about the song lead to the user’s interest in further discussion. After sharing the recruiter’s role with the study, the user chose to enroll in the study.

#### Inability to Track Profiles and Messages

Tracking contacts on *A4A* were not straightforward and required additional steps. *A4A* offers a feature to “favorite” users, allowing their profile to be bookmarked through an in-app list. This list enabled the recruiter to stay connected to contacts even if they changed their username. However, the feature did not allow for more than one person to be added owing to technical bugs. Thus, the recruiter used the web-based version to save the URLs of users’ profiles for tracking purposes. Additionally, the chat function only allows for a limited number of messages to be sent before older messages are lost. To save relevant information, the recruiter tracked and recorded usernames, dates of interaction, follow-up dates, user profile URLs, and other notes in Microsoft Excel.

#### Removal of Flyer Image From Recruiter Profile

During the recruitment process on *A4A*, the recruiter received an automated message indicating that the study’s flyer image—which had been uploaded to the recruiter’s profile—was removed because it violated the app’s standards. The recruiter then changed the study’s profile picture to a photo of himself. Thereafter, when potential participants expressed interest in the study through private SMS text messaging, the recruiter would send the flyer image directly to them.

#### Harassment

The recruiter experienced racially and politically charged verbal abuse during the height of the Black Lives Matter protests in 2020. Racial epithets (eg, “mountain caucus monkey”) were used by an app-user without provocation. Romantic and sexual harassment were common.

### Growlr

Similar to *A4A* and *Jack’d, Growlr* recruitment procedures involved both active and passive approaches. The study recruiter identified potential participants through the app’s search filters and messaged eligible users privately; in-app advertisements with the study information also contained a weblink to the prescreener. A *Growlr* paid feature “SHOUT!” was used to send the study information to multiple users in a specified region. We paid for “SHOUT!” broadcasts in 4 separate cities (eg, Charlotte and Raleigh, North Carolina; Nashville, Tennessee; and Cleveland, Ohio). Users within a 25-30–mile radius were able to see these advertisements and resulted in 2955 total views.

Similar to *A4A*, the recruiter experienced verbal abuse and harassment. *Growlr* removed the study flyer from the recruiter’s profile, with a message indicating that it violated company guidelines against in-platform solicitation. However, after a new flyer was posted that excluded any mention of the study participants being paid, it was nevertheless removed again, and *Growlr’s* customer service did not respond to our inquiries about the second flyer removal. Owing to the technical bugs and low success rate (no eligible couples were found), the recruiter discontinued efforts on the platform after 2 weeks.

## Discussion

### Principal Findings

HIV incidence among Black MSM in the United States continues to be disproportionately high [3,4] with one-third to half of Black HIV-positive MSM to be in a primary relationship [13,14,32]. Nonetheless, societal stigma, distrust of research and medical institutions, and other systemic barriers negatively impact HIV prevention and treatment for this underserved community [24,25]. As such, novel approaches to recruiting Black MSM couples are needed.

There are relatively few dyadic HIV research studies with Black MSM couples (eg, time and staffing). Little information exists detailing the successful strategies for web-based recruitment of Black MSM couples into HIV research. While dating and social networking apps have been commonly used to recruit single MSM for research studies [19], no research has used dating apps to explicitly recruit couples of MSM. This study demonstrated the feasibility of dating and social networking apps for recruiting Black MSM couples as part of a pilot RCT of a couples-focused app for improving HIV care engagement. Recruiting MSM couples through dating and social networking apps is a necessary recruitment strategy given the prevalence of sexual agreements among MSM couples [27].

Consistent with previous research, this sample of couples contained predominately same-race Black partnerships [32]. The search and filter functions in the apps, such as filtering users on the basis of their reported relationship status, helped to identify potential participants per the eligibility criteria. *A4A* and *Growlr* offered the functionality to filter through user-identified race or ethnicity categories, which reduced the time needed to search for eligible users. Paid advertising campaigns through *Jack’d* and *Growlr* were an opportunity to recruit passively, instead of actively searching through users and initiating conversations to determine eligibility and interest.

Although the strategy of privately messaging potential participants on *A4A* was a successful recruitment strategy, it was not without challenges. Our Black, same-gender-loving–identified recruiter reported multiple episodes of harassment of various types (eg, sexual, racial, and political). Additionally, app-specific guidelines for study advertisements varied (eg, character limits and other rules). Regular check-ins between the principal investigator and recruiters and careful attention to the guidelines for each app are necessary.

### Limitations

Our study recruited for a one-time interview, and we do not know how these findings generalize to other, longer-term research requirements. Further, biases in the sample skew toward nonmonogamous couples owing to the generally sexual purposes of MSM using the apps. Finally, given the evolving nature of the software, some of the app features reported during the time of publication may or may not reflect what is currently available.

### Comparison With Prior Work

Apps designed for MSM have become increasingly popular and users on those platforms may visit them frequently (eg, daily) [33]. Research has recruited single MSM [19,34-36] and Black MSM [37-39] via apps. Given high HIV transmission rates between MSM primary partners [8-11], recent studies have also recruited MSM couples through a combination of web-based engagement (eg, Facebook and gay websites) and apps [10,40-44], but not exclusively on apps. No research documents the utility of app-based recruitment for Black MSM couples [45,46]. Given the disproportionate rates of HIV [6,7,47] and the importance of coordinating HIV prevention, care, and treatment [45,48,49] within this community, there is urgency to finding novel approaches to recruiting Black MSM couples for HIV prevention studies.

### Conclusions

Dyadic HIV research with Black MSM couples is important but knowledge gaps remain. Challenges to research with this population include participant recruitment, which can be resource intensive, underscoring the need for recruitment strategies that have been demonstrated to be feasible and acceptable. We discuss our strategies for engaging Black MSM couples via social networking apps, and associated technical challenges, including issues with harassment directed at our recruiter. We have identified a way forward with using social networking apps to engage Black sexual-minority couples to inform future research.

## Abbreviations

A4A: Adam4Adam
MSM: men who have sex with men
RCT: randomized controlled trial
